# Supplementary material for: Birth month and infant gross motor development: Results from the Japan Environment and Children’s Study (JECS)
Source: PLoS One. 2021 May 20;16(5):e0251581. doi: 10.1371/journal.pone.0251581 (PMC8136702; doi:10.1371/journal.pone.0251581)
Supplement: S1 Table — (DOCX) [file pone.0251581.s001.docx]

**Table S1: Participant characteristics**

| Independent  variable |  | Total  n = 72,203 | ASQ at 6 mo Gross Motor score <22.25  n = 14,960 (20.7%) | *p* value | ASQ at 12 mo  Gross Motor  score <21.49  n = 10,260  (14.2%) | *p* value |
| --- | --- | --- | --- | --- | --- | --- |

| Daily time outside | |  |  |  | **<0.001** |  |  | **<0.001** |
| --- | --- | --- | --- | --- | --- | --- | --- | --- |
| None | | 13,091 | 2,960 | (22.6) |  | 2,051 | (15.7) |  |
| ≥ 1 h |  | 56,369 | 11,412 | (20.3) |  | 7,796 | (13.8) |  |
| Pre-pregnancy BMI | |  |  |  | 0.052 |  |  | **0.005** |
| <18.5 |  | 11,585 | 2,467 | (21.3) |  |  |  |  |
| ≥18.5 & <25 |  | 53,432 | 11,072 | (20.7) |  |  |  |  |
| ≥25 & <30 |  | 5,565 | 1,116 | (20.1) |  |  |  |  |
| ≥30 |  | 1,595 | 303 | (19.0) |  |  |  |  |
| Maternal age at birth | |  |  |  | **<0.001** |  |  | **<0.001** |
| <20 |  | 430 | 49 | (11.4) |  | 26 | ( 6.1) |  |
| ≥20 & <35 |  | 51,976 | 10,318 | (19.9) |  | 6,881 | (13.2) |  |
| ≥35 |  | 19,794 | 4,592 | (23.2) |  | 3,353 | (16.9) |  |
|  |  |  |  |  |  |  |  |  |
| Parity |  |  |  |  | **<0.001** |  |  | **0.003** |
| First time |  | 5,853 | 5,853 | (19.7) |  | 4,389 | (14.8) |  |
| Second time |  | 5,787 | 5,787 | (21.2) |  | 3,781 | (13.9) |  |
| Third or more |  | 2,993 | 2,993 | (22.2) |  | 1,838 | (13.6) |  |
| ART |  |  |  |  | **<0.001** |  |  | **<0.001** |
| no |  | 66,839 | 13,742 | (20.6) |  | 9,306 | (13.9) |  |
| yes |  | 5,077 | 1,159 | (22.8) |  | 913 | (18.0) |  |
| Threatened Abortion /  Premature labor | |  |  |  | **<0.001** |  |  | **<0.001** |
| no |  | 53,756 | 10,936 | (20.3) |  | 7,503 | (14.0) |  |
| yes |  | 18,447 | 4,024 | (21.8) |  | 2,757 | (15.0) |  |
| Neuropsychotropic drugs |  |  |  |  | 0.196 |  |  | **0.001** |
| no |  | 71,493 | 14,799 | (20.7) |  | 10,128 | (14.2) |  |
| yes |  | 710 | 161 | (22.7) |  | 132 | (18.6) |  |
| SGA |  |  |  |  | **<0.001** |  |  | **<0.001** |
| no |  | 65,040 | 13,323 | (20.5) |  | 9,132 | (14.0) |  |
| yes |  | 7,163 | 1,637 | (22.9) |  | 1,128 | (15.8) |  |
| Twins/Multiple birth |  |  |  |  | **<0.001** |  |  | **<0.001** |
| no |  | 71,473 | 14,722 | (20.6) |  | 10,119 | (14.2) |  |
| yes |  | 710 | 230 | (32.4) |  | 138 | (19.4) |  |
| Breech/Foot/Other abnormal presentation |  |  |  |  | **<0.001** |  |  | **<0.001** |
| no |  | 69,625 | 14,230 | (20.4) |  | 9,726 | (14.0) |  |
| yes |  | 2,578 | 730 | (28.3) |  | 534 | (20.7) |  |
| Induced delivery |  |  |  |  | **<0.001** |  |  | 0.130 |
| no |  | 58,964 | 12,419 | (21.1) |  | 8,448 | (14.3) |  |
| yes |  | 13,071 | 2,507 | (19.2) |  | 1,786 | (13.7) |  |
| C-section |  |  |  |  | **<0.001** |  |  | **<0.001** |
| no |  | 59,111 | 11,557 | (19.6) |  | 7,886 | (13.3) |  |
| yes |  | 12,924 | 3,369 | (26.1) |  | 2,348 | (18.2) |  |
| Labor >24 h |  |  |  |  | **<0.001** |  |  | **<0.001** |
| no |  | 69,745 | 14,536 | (20.8) |  | 9,950 | (14.3) |  |
| yes |  | 2,458 | 424 | (17.3) |  | 310 | (12.6) |  |

| Independent  variable |  | Total  n = 72,203 | ASQ at 6 mo Gross Motor score <22.25  n = 14,960 (20.7%) | *p* value | ASQ at 12 mo  Gross Motor  score <21.49  n = 10,260  (14.2%) | *p* value |
| --- | --- | --- | --- | --- | --- | --- |

(Table S1: Continued)

| Apgar score (5 min) |  |  |  |  | **<0.001** |  |  | **<0.001** |
| --- | --- | --- | --- | --- | --- | --- | --- | --- |
| ≥9 |  | 65,823 | 13,526 | (20.6) |  | 9,206 | (14.0) |  |
| <9 |  | 3,088 | 728 | (23.6) |  | 577 | (18.7) |  |
| Cord/Placenta problems |  |  |  |  | **0.025** |  |  | 0.082 |
| no |  | 53,986 | 11,314 | (21.0) |  | 7,684 | (14.2) |  |
| yes |  | 16,213 | 3,247 | (20.0) |  | 2,260 | (13.9) |  |
| Meconium staining |  |  |  |  | **<0.001** |  |  | **0.002** |
| no |  | 69,788 | 14,533 | (20.8) |  | 9,969 | (14.3) |  |
| yes |  | 2,415 | 427 | (17.7) |  | 291 | (12.1) |  |
| Neonatal transportation | |  |  |  | **<0.001** |  |  | **<0.001** |
| no |  | 67,602 | 13,880 | (20.5) |  | 9,400 | (13.9) |  |
| yes |  | 2,757 | 716 | (26.0) |  | 591 | (21.4) |  |
| Respiratory distress |  |  |  |  | 0.205 |  |  | **<0.001** |
| no |  | 70,663 | 14,621 | (20.7) |  | 9,968 | (14.1) |  |
| yes |  | 1,540 | 339 | (22.0) |  | 292 | (19.0) |  |
| Hyperbilirubinemia |  |  |  |  | **<0.001** |  |  |  |
| no |  | 65,250 | 13,403 | (20.5) |  | 9,221 | (14.1) |  |
| yes |  | 6,953 | 1,557 | (22.4) |  | 1,039 | (14.9) |  |
| IUGR |  |  |  |  | **<0.001** |  |  | **<0.001** |
| no |  | 70,973 | 14,612 | (20.6) |  | 10,024 | (14.1) |  |
| yes |  | 1,230 | 348 | (28.3) |  | 236 | (19.2) |  |
| Smoking during pregnancy |  |  |  |  | **<0.001** |  |  | **<0.001** |
| no |  | 69,137 | 14,415 | (20.9) |  | 9,933 | (14.4) |  |
| yes |  | 2,563 | 450 | (17.6) |  | 268 | (10.5) |  |
| Drinking during pregnancy |  |  |  |  | 0.296 |  |  | **<0.001** |
| no |  | 64,728 | 13,374 | (20.7) |  | 9,085 | (14.0) |  |
| yes |  | 7,196 | 1,534 | (21.3) |  | 1,140 | (15.8) |  |
| EPDS |  |  |  |  | **<0.001** |  |  | **0.004** |
| <9 |  | 63,508 | 13,046 | (20.5) |  | 8,956 | (14.1) |  |
| ≥9 |  | 7,710 | 1,718 | (22.3) |  | 1,182 | (15.3) |  |
| Education |  |  |  |  | **0.013** |  |  | **<0.001** |
| Junior High/High |  | 24,615 | 4,963 | (20.2) |  | 3,213 | (13.1) |  |
| College/Vocational |  | 30,718 | 6,453 | (21.0) |  | 4,375 | (14.2) |  |
| University/Graduate |  | 16,555 | 3,464 | (20.9) |  | 2,631 | (15.9) |  |
| Income |  |  |  |  | 0.756 |  |  | **<0.001** |
| <4 mil yen |  | 26,331 | 5,428 | (20.6) |  | 3,546 | (13.5) |  |
| ≥4 mil & <6 mil yen |  | 22,629 | 4,666 | (20.6) |  | 3,246 | (14.3) |  |
| ≥6 mil & <10 mil yen |  | 15,647 | 3,258 | (20.8) |  | 2,351 | (15.0) |  |

| Abbreviations: ART, Assisted Reproductive Treatments; EPDS, Edinburgh Postnatal Depression Scale; SGA, Small for Gestational Age; IUGR, Intrauterine Growth Restriction.  *Variables not included in this table with *p* values ≥ 0.05: prenatal dietary intake of vitamin D, prenatal vitamin D supplement, prenatal usage of sunscreen, history of spontaneous abortion, antibiotics during pregnancy, hypertension/PIH, psychiatric specialist care during pregnancy, diabetes/GDM, maternal epilepsy, maternal thyroidism, suction/forceps extraction, epidural, and exclusive breastfeeding for the first six months. |
| --- |
|  |
|  |
|  |
