# Supplementary material for: Birth month and infant gross motor development: Results from the Japan Environment and Children’s Study (JECS)
Source: PLoS One. 2021 May 20;16(5):e0251581. doi: 10.1371/journal.pone.0251581 (PMC8136702; doi:10.1371/journal.pone.0251581)
Supplement: Attachment — Submitted filename: Comments.docx [file pone.0251581.s002.docx]

Thank you for the opportunity to review the manuscript. The manuscript is very clearly organized and well written. It presents findings moderately contributing to our understanding of the relationship between birth month and gross motor development of young child, using a large sample data. I have a few comments regarding different sections of the manuscript.

**Introduction.**

The study needs more information on previous findings on relationship between birth month and gross motor skills in early childhood, even the previous findings were not from large cohort samples. What has the literature found? Has the literature found significantly positive/negative relationship between the two variables of interest? Has any literature investigated the trajectories of motor development of young children? I think it needs to add more motivations of the study in this section.

**Methods.**

1. What’s the strategies of the sampling? What’s the process of data collection? Such as data on vitamin D intake, etc. Readers may want to know more detailed information on sampling and data collection.
2. As the measurement of child gross motor development, how about the internal consistency of the ASQ-3 for the sample used in the study? I would suggest the authors reporting this in the methods section.

**Results.**

1. Table 1 looks too big. And it was inserted in the wrong place of the manuscript. It is also true for Tables 2 & 3.
2. On line 173, a quotation mark has been missed.

**Discussion.**

1. Again, more literature should be revisited in this section regarding to the issues that are discussed here.
2. What are the implications of the study? The implications should be discussed here or in the conclusion section.
