# Supplementary material for: Birth month and infant gross motor development: Results from the Japan Environment and Children’s Study (JECS)
Source: PLoS One. 2021 May 20;16(5):e0251581. doi: 10.1371/journal.pone.0251581 (PMC8136702; doi:10.1371/journal.pone.0251581)
Supplement: Attachment — Submitted filename: Response to Reviewers.docx [file pone.0251581.s003.docx]

18 March, 2021

**Responses to Editor’s letter (in word file) and comments by Reviewer 2 (in email)**

The comments in the attached word file (from the Editor) and Reviewer 2’s comments in the email seemed to be identical. Therefore, we provided the same responses in both sections (just below and the last two pages of this file).

All our responses were in blue letters.

**Introduction.**

The study needs more information on previous findings on relationship between birth month and gross motor skills in early childhood, even the previous findings were not from large cohort samples. What has the literature found? Has the literature found significantly positive/negative relationship between the two variables of interest? Has any literature investigated the trajectories of motor development of young children? I think it needs to add more motivations of the study in this section.

Thank you for your suggestion to improve the introduction. We added more information on results of previous studies (LL54-69).

.

**Methods.**

1. What’s the strategies of the sampling? What’s the process of data collection? Such as data on vitamin D intake, etc. Readers may want to know more detailed information on sampling and data collection.

We added some more details on sampling of the JECS, referring to the protocol paper and a paper on the basic characteristics of the JECS subjects. Further, we added information on the baseline characteristics, which were comparable to the national survey (LL81-86, LL100-103).

2. As the measurement of child gross motor development, how about the internal consistency of the ASQ-3 for the sample used in the study? I would suggest the authors reporting this in the methods section.

Cronbach’s alpha (0.76 for 6m and 0.78 for 12m) was added to the Method section (LL 113-115).

**Results.**

1. Table 1 looks too big. And it was inserted in the wrong place of the manuscript. It is also true for Tables 2 & 3.

Our apology for inserting them into the wrong places. They are all in the results section. Table 1 has been shortened with the information only on birth month and region/latitude. The rest of the old Table1 was removed now as they were used only for adjusting the models. (We have created Table S1 and uploaded it separately from the manuscript in case the information on the adjusting variables is necessary.)

2. On line 173, a quotation mark has been missed.

Apology for the mistake. The quotation is added now (LL248-249).

**Discussion.**

1. Again, more literature should be revisited in this section regarding to the issues that are discussed here.

We added some literature to strengthen our argument (Please see the second paragraph of Discussion (LL252-262).

2. What are the implications of the study? The implications should be discussed here or in the conclusion section.

Following your suggestion, in addition to the second sentence from the last, we changed the final sentence as follows (LL 374-380) :

“While further follow-up on this JECS cohort will confirm the long-term influence of birth month on child neuromotor development, an important clinical implication of the present study is that seasonality during the prenatal and neonatal periods needs to be incorporated in clinical research to properly assess the lifetime health of offspring.“

**Additional Editor Comments:**

We have received comments on your manuscript from two reviewers. I also have additional comments on your paper. Please address all comments carefully in your revised manuscript.

Comments:

1) Please clearly justify the cut-off values you have used for the neurodevelopmental scores.

Thank you for your comment. We used the cut-off values, following ASQ-3 manual, and made some changes in the manuscript so that it is clearer for the readers. (LL117-121).

2) You need to state how you dealt with missing information. Specifically, I assume that there was missing information in at least some of the covariates you adjusted for in the multivariable analysis. If there was no missing in the covariates, then this should be stated. If there was missing information, then you should consider multiple imputation.

Thank you for your suggestion. As you pointed out, 15 covariates, out of 24 covariates to adjust the model, had some missing values. We conducted Multiple Imputation by Chained Equation, in which the above-mentioned 15 variables with missing values were imputed by 13 variables with completed values (9 covariates plus gross motor development score at 6 and 12 months, birth month, and latitude). As the results were very similar between those without imputation, we stated that imputed results were not different and used those without imputation. Please see the table below for the details. (LL140-142)

3) Some of the covariates you adjusted for are likely to be mediators (potentially part of the explanatory pathway) and not confounders. I am for example thinking of all the pregnancy outcomes which you adjust for. You should clearly classify which covariates are confounders as opposed to potential mediators. You should also not adjust your main model for the potential mediators but include these in a second multivariable model.

Thank you very much for the very important point. We fully agree that all the pregnancy outcomes could be potential mediators, and conducted Poisson regression analysis, with birth month as outcome and each pregnancy-related variable as exposure. The variables assessed for potential mediators were: prepreganancy BMI; maternal age; parity; Assisted Reproductive Treatments (ART); threatened abortion/premature labor; neuropsychotropic drugs; smallness for gestational age; multiple birth; breech/foot/other abnormal presentation; induced delivery; C-section; labor>24h; Apgar Score (5 min); umbilical cord/placenta problems; meconium staining; neonatal transportation; respiratory distress; hyperbilirubinemia; Intrauterine Growth Restriction; and Edinburgh Postnatal Depression Scale (EPDS) at 6months.

*P*-values of most variables except 7 variables (maternal age, ART, C-section, umbilical cord/placenta problems, meconium staining, hyperbilirubinemia, EPDS) were larger than 0.05. Even though these 7 variables showed *p*<0.05, Relative Risks were just around 1.00, with extremely narrow 95%CI just below or above 1.00.

Finally, we created a new model without the above-written 7 potential mediators at each time point (at 6 and 12 months of age. Please see the gray shady areas). As the aRR values, *p* values, and the overall trend of aRR were very similar between the two different models – with/without 7 potential mediators both at 6 and 12 months of age –, we decided to keep the original adjusted models, with all the covariates including the 7 variables. Please see the table below.

4) I wonder why you chose to look at season of delivery and not season of conception? Since you mention that intra uterine vitamin D levels during the first trimester might be part of the explanatory mechanism, I wonder if season of conception is not of more interest? Please carefully think this through and justify your decision.

Thank you very much for your comment on the importance of focusing on the timing of conception. We absolutely agree with your point. However, very unfortunately, only birth month data were available for the analyses. Information on conception dates was not recorded, and although the birthdays were recorded, the Ministry of the Environment did not provide it with the researchers to protect the privacy of the participants. As there could be maximum thirty-day difference within the same birth month, it is not accurate to estimate the timing of conception from birth month and gestational age. As a result, we decided to utilize birth month information for this paper.

As one of the inclusion criteria is full-term delivery for this study and approximately 80% of the participants were born week 38-40, therefore, their conception seasons could be not so widely spread if they were born in the same month. Thus, by looking at birth season, we could also discuss about the other side of the coin, the conception season.

5) Move all results from the methods to the results section. There should be no results tables or references to these in the methods.

They are now moved to the results section (Our apology for inserting them wrongly.)

6) The manuscript would greatly benefit from language editing. I strongly suggest you consider sending the manuscript for professional language editing.

The English in this manuscript was carefully corrected by two professional editors, both with extensive research editing experience. One of these editors is a native English speaker. In addition, a native English speaker in our institute and one of our co-authors, a native English speaker, have also proof-read it.

7) Please clarify why you chose to use poission regression (IRR) instead of a log-binomial regression (RR)?

Both log-binomial model and modified Poisson regression model produce relative risk with similar magnitude, and previous studies showed that modified Poisson regression has been proved to be as reliable for analyses of prospective studies.(1-3) In our data set, log-binomial models did not converge to provide parameter estimates. Therefore, we estimated relative risk by using the modified Poisson regression model with a robust variance estimator.

1.	Zou G. A modified poisson regression approach to prospective studies with binary data. Am J Epidemiol. 2004;159(7):702-6.

2.	Yelland LN, Salter AB, Ryan P. Performance of the modified Poisson regression approach for estimating relative risks from clustered prospective data. Am J Epidemiol. 2011;174(8):984-92.

3.	Chen W, Qian L, Shi J, Franklin M. Comparing performance between log-binomial and robust Poisson regression models for estimating risk ratios under model misspecification. BMC Med Res Methodol. 2018;18(1):63.

Journal Requirements:

When submitting your revision, we need you to address these additional requirements.

1.) Please ensure that your manuscript meets PLOS ONE's style requirements, including those for file naming. The PLOS ONE style templates can be found at

<https://journals.plos.org/plosone/s/file?id=wjVg/PLOSOne_formatting_sample_main_body.pdf> and

<https://journals.plos.org/plosone/s/file?id=ba62/PLOSOne_formatting_sample_title_authors_affiliations.pdf>

Thank you for the instructions. We have read them and believe that we have followed the points.

2.) Thank you for including your ethics statement:  "The JECS protocol was reviewed and approved by the Ministry of the Environment’s Institutional Review Board on Epidemiological Studies and by the Ethics Committees of all participating institutions. Written informed consent was obtained from all participating women and men. The below link contains the JECS ethical information (p17)
<http://www.nies.go.jp/kanko/news/38/38-4/jqjm1000000i2db3-att/jqjm1000000i2dc0.pdf>".

Please amend your current ethics statement to include the full name of the ethics committee/institutional review board(s) that approved your specific study.

Once you have amended this/these statement(s) in the Methods section of the manuscript, please add the same text to the “Ethics Statement” field of the submission form (via “Edit Submission”).

For additional information about PLOS ONE ethical requirements for human subjects research, please refer to <http://journals.plos.org/plosone/s/submission-guidelines#loc-human-subjects-research>.

The full name of the ethics committee/institutional review boards were added both to the manuscript (LL151-156) and the PLOS ONE “Ethics Statement” field.

3.) In statistical methods, please clarify whether you corrected for multiple comparisons.

Thank you for your comment. For the analysis for Table2, as we assessed the association between birth month and gross motor development at 6 months and 12 months separately, there was no need to conduct multiple comparison.

For Table 3, we divided the subjects into 4 groups (BEST, IMPROVED, WORSENED, and WORST) on the basis of the frequency of those who did not meet the cut-off values of the ASQ scores at 6 months and 12 months. As we assessed the frequency of those who did not meet the cut-off (not mean of the ASQ scores), we did not have to correct for multiple comparison. Therefore, we did not state about multiple comparisons in the Statistical Analysis section.

4.) Thank you for stating the following in the Acknowledgments Section of your manuscript:

'The Japan Environment and Children’s Study was funded by the Ministry of Environment, Japan.'

We note that you have provided funding information that is not currently declared in your Funding Statement. However, funding information should not appear in the Acknowledgments section or other areas of your manuscript. We will only publish funding information present in the Funding Statement section of the online submission form.

Please remove any funding-related text from the manuscript and let us know how you would like to update your Funding Statement. Currently, your Funding Statement reads as follows:

'The authors received no specific funding for this work.' Please include your amended statements within your cover letter; we will change the online submission form on your behalf.

Apology for the mistake. Following your instruction, the sentence was removed from the manuscript. Please change our Funding Statement as “The Japan Environment and Children’s Study is funded by the Ministry of the Environment, Japan”. (This statement is also included in the second paragraph of our cover letter.)

5.) One of the noted authors is a group or consortium Japan Environment and Children’s Study Group. In addition to naming the author group, please list the individual authors and affiliations within this group in the acknowledgments section of your manuscript. Please also indicate clearly a lead author for this group along with a contact email address.

We believe the list in Acknowledgement is what you pointed out above, including each individual authors and affiliations within this group. We updated the list as it was changed since we submitted our manuscript in October 2020 (LL 391-404). The original list did not contain PI (Prof. Michihiro Kamijima)’s email account, which is now added to the front page. (LL19-20).

**Reviewers' comments:**

Reviewer's Responses to Questions

**Comments to the Author**

1. Is the manuscript technically sound, and do the data support the conclusions?

The manuscript must describe a technically sound piece of scientific research with data that supports the conclusions. Experiments must have been conducted rigorously, with appropriate controls, replication, and sample sizes. The conclusions must be drawn appropriately based on the data presented.

Reviewer #1: Yes

Reviewer #2: Yes

2. Has the statistical analysis been performed appropriately and rigorously?

Reviewer #1: Yes

Reviewer #2: Yes

3. Have the authors made all data underlying the findings in their manuscript fully available?

The [PLOS Data policy](http://www.plosone.org/static/policies.action#sharing) requires authors to make all data underlying the findings described in their manuscript fully available without restriction, with rare exception (please refer to the Data Availability Statement in the manuscript PDF file). The data should be provided as part of the manuscript or its supporting information, or deposited to a public repository. For example, in addition to summary statistics, the data points behind means, medians and variance measures should be available. If there are restrictions on publicly sharing data—e.g. participant privacy or use of data from a third party—those must be specified.

Reviewer #1: Yes

Reviewer #2: Yes

4. Is the manuscript presented in an intelligible fashion and written in standard English?

PLOS ONE does not copyedit accepted manuscripts, so the language in submitted articles must be clear, correct, and unambiguous. Any typographical or grammatical errors should be corrected at revision, so please note any specific errors here.

Reviewer #1: Yes

Reviewer #2: Yes

5. Review Comments to the Author

Please use the space provided to explain your answers to the questions above. You may also include additional comments for the author, including concerns about dual publication, research ethics, or publication ethics. (Please upload your review as an attachment if it exceeds 20,000 characters)

Reviewer #1: Abstract:
-It is appropriate to mentioned P values in significant result.

Thank you for your comments. Following your comments, we re-write the sentence as follows “Summer-born infants had the worst outcomes at both 6 months and 12 months of age. This outcome applied to the ASQ-3 score itself and to the adjusted Relative Risk (aRR), with the highest aRRs (relative to January-born) among August-born (aRR 2.51; 95%CI 2.27-2.78 at 6 months), and June-born (aRR 1.84; 95%CI 1.63-2.09 at 12 months)”

As the sentence is in abstract and we would like to keep it as concise as possible, we stated 95%CI, instead of *p* values, of the peak month for each time point. (LL 32-35)

Methods:
-The following phrase, Fig 1, Table 1, 2 and 3 which is given in the statistical analysis section, should be given in the results section. “The means of maternal age at enrolment for the study subjects and 81 the excluded were 30.8 (95% CI: 30.0, 30.9) and 30.3 (95% CI: 30.2, 30.3) years old”

Our apology for the mistakes. The figures and tables were moved to the results section, so was the sentence. (LL 160-162)

Discussion:
In methods section mentioned:

-“The questionnaires covered various topics, including lifestyle, socio-economic status (SES), diet, medical history, and medication”.

And in Table 1, many independent variables were mentioned in the study cases, such as prepregnancy BMI, hyperbilirubinemia, Apgar score, C-section, which there were significant difference. But in the discussion, there is no mention of the possibility of the effect of the independent variables.

While appreciating your interest in other putative risk factors, it is, unfortunately, impossible for us to state RRs for other putative risk factors as we are not allowed/approved to do so withing the JECS system. In our JECS system, in order to avoid collision and confusion, every researcher needs to apply for their theme prior to writing a manuscript and to acquire an approval by the JECS National Centre. As the independent variables you pointed out do not belong to this particular paper, we cannot discuss into the details. As the focus of this paper is birth month, we do hope that what we currently provide in Discussion satisfy readers.

Some abbreviations need to be mentioned in full wherever they are first mentioned: EPDS, MR, LD.
Figure 2 is not clear.

Thank you very much for your suggestion. EPDS, MR, and LD are stated in full in the first appearance.

Both Figure 1 and 2 are now corrected by PACE, the recommended site by the editor.

Reviewer #2: Introduction.

The study needs more information on previous findings on relationship between birth month and gross motor skills in early childhood, even the previous findings were not from large cohort samples. What has the literature found? Has the literature found significantly positive/negative relationship between the two variables of interest? Has any literature investigated the trajectories of motor development of young children? I think it needs to add more motivations of the study in this section.
Thank you for your suggestion to improve the introduction. We added more information on results of previous studies (LL54-69).

Methods.
1. What’s the strategies of the sampling? What’s the process of data collection? Such as data on vitamin D intake, etc. Readers may want to know more detailed information on sampling and data collection.

We added some more details on sampling of the JECS, referring to the protocol paper and a paper on the basic characteristics of the JECS subjects. Further, we added information on the baseline characteristics, which were comparable to the national survey (LL81-86, LL100-103).

2. As the measurement of child gross motor development, how about the internal consistency of the ASQ-3 for the sample used in the study? I would suggest the authors reporting this in the methods section.

Cronbach’s alpha (0.76 for 6m and 0.78 for 12m) was added to the Method section (LL 113-115).

Results.
1. Table 1 looks too big. And it was inserted in the wrong place of the manuscript. It is also true for Tables 2 & 3.

Our apology for inserting them into the wrong places. They are all in the results section. Table 1 has been shortened with the information only on birth month and region/latitude. The rest of the old Table1 was removed now as they were used only for adjusting the models. (We have created Table S1 and uploaded it separately from the manuscript in case the information on the adjusting variables is necessary.)

2. On line 173, a quotation mark has been missed.

Apology for the mistake. The quotation is added now (LL248-249).

Discussion.

1. Again, more literature should be revisited in this section regarding to the issues that are discussed here.

We added some literature to strengthen our argument (Please see the second paragraph of Discussion (LL252-262).

2. What are the implications of the study? The implications should be discussed here or in the conclusion section.

Following your suggestion, in addition to the second sentence from the last, we changed the final sentence as follows (LL 374-380) :

“While further follow-up on this JECS cohort will confirm the long-term influence of birth month on child neuromotor development, an important clinical implication of the present study is that seasonality during the prenatal and neonatal periods needs to be incorporated in clinical research to properly assess the lifetime health of offspring.“

6. PLOS authors have the option to publish the peer review history of their article ([what does this mean?](https://journals.plos.org/plosone/s/editorial-and-peer-review-process#loc-peer-review-history)). If published, this will include your full peer review and any attached files.

If you choose “no”, your identity will remain anonymous but your review may still be made public.

**Do you want your identity to be public for this peer review?** For information about this choice, including consent withdrawal, please see our[Privacy Policy](https://www.plos.org/privacy-policy).

Reviewer #1: No

Reviewer #2: No

[NOTE: If reviewer comments were submitted as an attachment file, they will be attached to this email and accessible via the submission site. Please log into your account, locate the manuscript record, and check for the action link "View Attachments". If this link does not appear, there are no attachment files.]

While revising your submission, please upload your figure files to the Preflight Analysis and Conversion Engine (PACE) digital diagnostic tool, <https://pacev2.apexcovantage.com/>. PACE helps ensure that figures meet PLOS requirements. To use PACE, you must first register as a user. Registration is free. Then, login and navigate to the UPLOAD tab, where you will find detailed instructions on how to use the tool. If you encounter any issues or have any questions when using PACE, please email PLOS at [figures@plos.org](mailto:figures@plos.org). Please note that Supporting Information files do not need this step.

Thank you for the information. I PACE-corrected both figures.

*In compliance with data protection regulations, you may request that we remove your personal registration details at any time.* [*(Remove my information/details)*](https://www.editorialmanager.com/pone/login.asp?a=r)*. Please contact the publication office if you have any questions.*
